# Electrocardiographic markers of hypertrophy in children: revisiting R/S overlap and the Katz-Wachtel phenomenon

**DOI:** 10.3389/fped.2025.1713053

**Published:** 2025-12-04

**Authors:** Nikola Owsianka, Lukas Schober, Hannes Sallmon, Daniel Scherr, n Köstenberger, Martin Manninger, Nathalie Öffl, Stefan Kurath-Koller

**Affiliations:** 1 Division of Pediatric Cardiology, Department of Pediatrics and Adolescence Medicine, Medical University Graz, Graz, Austria; 2 Division of Cardiology, Department of Medicine, Medical University Graz, Graz, Austria

**Keywords:** ECG, children, ventricular hypertrophy, chest leads, Katz-Wachtel-Phenomenon, biventricular hypertrophy, electrocardiography

## Abstract

**Background:**

Many children show R/S overlap in ECG chest leads. The Katz-Wachtel phenomenon, described in the 1960s, characterizes high R and S waves ≥50 mm as an ECG feature of biventricular cardiac hypertrophy. This study investigates the KWP and the association between an R/S overlap and cardiac hypertrophy.

**Methods:**

Retrospective study including 800 pediatric patients, treated at the Division of Pediatric Cardiology, University Hospital Graz, between 2016 and 2022. ECG measurements with regard to R/S overlap and voltage in chest leads and review of echocardiography findings of cardiac hypertrophy. ECG measurements were manually obtained and reviewed by two blinded pediatric cardiologists.

**Results:**

Of the 500 patients with an R/S overlap, 4% had confirmed cardiac hypertrophy compared to 1% of children without confirmed R/S overlap. Sensitivity was highest in leads V3/4 (69%), while specificity was highest in leads V1/V2 (93%) and V5/V6 (96%), respectively. A statistically significant correlation between R/S overlap and hypertrophy was found.

**Conclusion:**

R/S overlap correlates with myocardial hypertrophy in children. While it demonstrates high sensitivity, its limited specificity prevents its use as a standalone diagnostic parameter. It may contribute to early identification when combined with other ECG features and echocardiographic evaluation.

## Impact

- R/S overlap in chest leads correlates with ventricular hypertrophy in children
- The Katz-Wachtel Phenomenon should not be considered in the current era of digital ECG recording
- R/S overlap should be used for screening purposes but not diagnosis of ventricular hypertrophy
- R/S overlap should prompt echocardiography

## Introduction

Cardiac hypertrophy in children, whether secondary to congenital heart disease or acquired conditions, poses a significant risk for complications such as heart failure, arrhythmias, and sudden cardiac death. Early detection is critical, and electrocardiography (ECG) remains a widely used, non-invasive, and accessible tool in pediatric cardiology for initial assessment and monitoring of ventricular hypertrophy (1).

Despite its routine use, interpreting ECGs in children presents unique challenges due to age-dependent changes in cardiac anatomy, heart rate, and electrical conduction patterns. Established ECG criteria for diagnosing left or right ventricular hypertrophy—such as increased QRS voltages or axis deviations—are primarily derived from adult populations and may not translate directly to pediatric physiology (2).

Two ECG patterns historically associated with ventricular hypertrophy in children are R/S overlap in precordial leads (3) and the Katz-Wachtel phenomenon (KWP) (4). R/S overlap refers to the persistence of both prominent R and S waves across adjacent chest leads, potentially indicating biventricular involvement. The Katz-Wachtel phenomenon, first described in the early 20th century, is characterized by high-amplitude biphasic QRS complexes and has long been cited as a marker of severe biventricular hypertrophy, particularly in neonates and infants with congenital heart disease (4–7). While frequently referenced in teaching materials, these patterns have not been systematically validated in contemporary pediatric populations, and their relevance in the era of digital ECG interpretation is uncertain (8–12).

The aim of this study is to critically evaluate the diagnostic performance and clinical utility of R/S overlap and the Katz-Wachtel phenomenon in a large, real-world pediatric cohort. By analyzing their prevalence, sensitivity, specificity, and association with echocardiographically confirmed cardiac hypertrophy, we seek to clarify the diagnostic value of these traditional ECG markers and their role in modern pediatric cardiology.

## Methods

### Study design

This retrospective cohort study was conducted and carried out at the Division of Pediatric Cardiology, Department of Pediatrics and Adolescent Medicine, University Hospital Graz, Austria, and included patients treated at the outpatient ward between January 2016 and December 2022.

### Patient population

**Inclusion Criteria:**

- Patients from birth to 18 years of age.
- Patients who underwent ECG and echocardiographic evaluation at the Division of Pediatric Cardiology, University Hospital Graz.
- Availability of complete medical records, including ECG and echocardiographic data.

**Exclusion Criteria:**

- Patients with congenital heart defects other than ventricular hypertrophy.
- Patients with metabolic or systemic diseases affecting myocardial tissue.

ECG and echocardiographic examinations were obtained as part of the patients' routine diagnostic work-up in our pediatric cardiology department. The most common clinical indications included evaluation of cardiac murmurs, syncope, accidental electrical injuries, and known congenital heart defects such as atrial septal defect (ASD II), ventricular septal defect (VSD), or patent foramen ovale (PFO). Additional reasons for referral included screening due to a family history of sudden cardiac death or cardiomyopathy, evaluation of systemic or genetic conditions with potential cardiac involvement (e.g., trisomy 21, cystic fibrosis, sickle cell disease, Marfan syndrome), and follow-up after cardiac or systemic conditions such as Kawasaki disease, kidney transplantation, or previous chemotherapy for malignancy.

### Data collection

#### Electronic medical records

Data were retrospectively collected using the electronic medical record system (MEDOCS, SAP, Walldorf, Germany). Information gathered included demographic data (age, sex, weight, height), ECG measurements (KISS ECG recording system, GE Healthcare, Chicago, IL, USA), and echocardiographic findings (Philips cardiovascular ultrasound EPIQ Elite; Philips, Amsterdam, Netherlands).

For each patient, only a single ECG—the most recent standard 12-lead ECG available during the study period—was included in the analysis. No patient was included more than once, and serial or follow-up ECGs were not considered. ECGs were reviewed using digitally stored recordings (KISS ECG system, GE Healthcare) via electronic workstations.

#### ECG measurements

**Figure 1:**
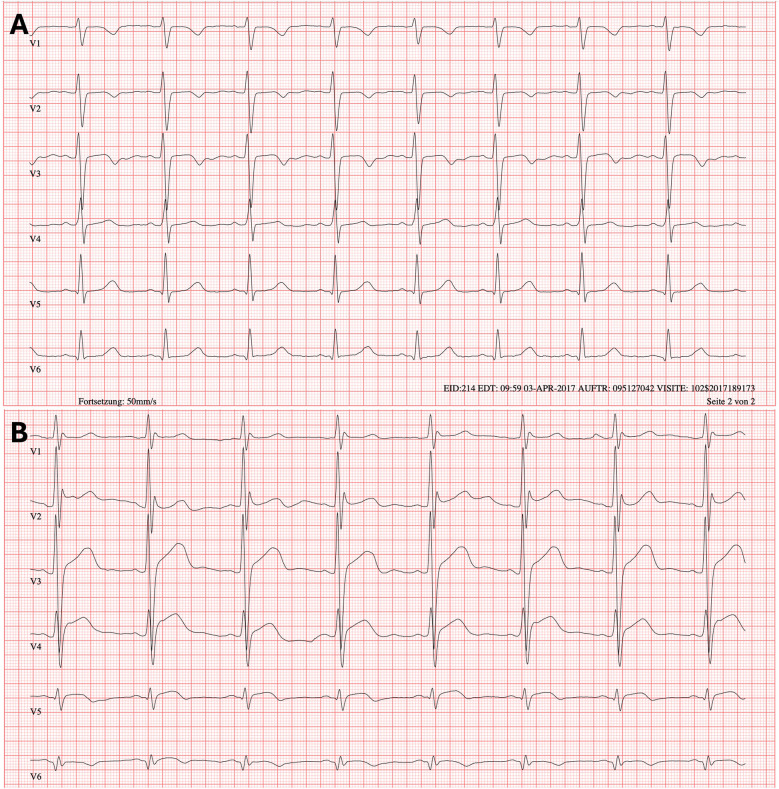
**(A)** ECG example of R/S overlap V 3/4. **(B)** ECG example of Katz-Wachtel-Phenomenon. Paper speed 50 mm/s, scaling 10 mm/mV; ECG leads are given a schest leads V1–V6.

ECGs were thoroughly assessed with regard to measurements of P wave duration (ms) and amplitude (mV), PQ interval (ms), QRS complex duration (ms), QT interval and QTc (using Bazett's formula; ms), Amplitudes of the R and S waves in chest leads V1 to V6 (mV), R/S ratio for each chest lead, R/S overlap in chest leads, and KWP criteria. R/S overlap was defined as an overlap of at least 0.1 mm (i.e., 0.1 mV) between the R and S waves of two adjacent chest leads (V1–V6) in a standard 12-lead ECG recording (1 mV = 10 mm; paper speed: 50 mm/s). The Katz-Wachtel phenomenon (KWP) was defined as a QRS voltage ≥50 mm (i.e., ≥5.0 mV) in any chest lead (V1–V6) under the same recording conditions. ECG examples are given in Figure 1.

R/S ratios and wave amplitudes were collected for exploratory and descriptive purposes only, and were not used as part of the diagnostic definition of hypertrophy.

#### Echocardiographic data

Echocardiography assessments were reviewed in every patient with regard to evidence of cardiac hypertrophy. Review of echocardiography images and cine-loops was performed by an experienced pediatric cardiologist. Parameters evaluated included: left ventricular mass (LVM, gramm), left ventricular posterior wall thickness (LVPW; mm), interventricular septal thickness (IVS; mm), presence of biventricular hypertrophy, and *z*-scores adjusted for body surface area (13).

### Statistical analysis

Statistical analyses were performed using SPSS (Statistical Package for the Social Sciences (IBM SPSS Statistics 30.0). Descriptive statistics, including means, standard deviations, and percentages, were calculated for demographic data, ECG parameters, and echocardiographic findings.

- Chi-square tests were used to assess the association between R/S overlap and the presence of cardiac hypertrophy. A 95% confidence interval was applied, and a *p*-value of <0.05 was considered statistically significant.
- Phi and Cramer's V values were calculated to determine the strength of the association between R/S overlap and cardiac hypertrophy. *T*-tests and analysis of variance (ANOVA) were used to compare the means of ECG parameters between different groups (e.g., with and without R/S overlap, with and without hypertrophy).
- The sensitivity and specificity of R/S overlap in detecting cardiac hypertrophy were calculated for each chest lead (V1–V6).
- ROC (Receiver Operating Characteristic) curves were generated to evaluate the diagnostic performance of R/S overlap and the Katz-Wachtel phenomenon in identifying cardiac hypertrophy.
- Inter-observer agreement for manual ECG measurements was evaluated by two independent pediatric cardiologists. For continuous variables (e.g., QT interval, QRS duration), intraclass correlation coefficients (ICC, two-way random effects, absolute agreement) were calculated. For binary classifications (presence of R/S overlap and Katz-Wachtel phenomenon), inter-rater agreement was assessed using Cohen's *κ* statistic.

### Ethical considerations

The study was approved by the Ethics Committee of the Medical University of Graz (EK Nr. 33-045 ex 20/21). Patient confidentiality and data protection were maintained throughout the study.

## Results

The study cohort consisted of 800 pediatric patients (mean age 8.5 ± 5.8 years), of which 359 (45%) were female. The R/S Overlap Group consisted of 500 (62.5%) patients with R/S overlap in at least one chest lead. The Non-Overlap Group consisted of 300 (37.5%) patients. Cardiac hypertrophy was confirmed by echocardiography in 23 patients (2.9%) of the total cohort. In the R/S Overlap Group, 20 patients (4%) had proven cardiac hypertrophy, two of which showed biventricular hypertrophy. In the Non-Overlap Group, 3 patients (1%) had proven cardiac hypertrophy, none biventricular. The overall sensitivity of R/S overlap for detecting cardiac hypertrophy was 87%, while the specificity was 38%. In addition to sensitivity and specificity, we calculated the positive predictive value (PPV) and negative predictive value (NPV) for both ECG criteria. For R/S overlap, the PPV was 4.0% and the NPV was 99.0%, reflecting its high ability to rule out hypertrophy when absent, but limited ability to confirm it when present. For the Katz-Wachtel phenomenon, the PPV was 0.0%, while the NPV was 97.1%, consistent with its poor sensitivity and extremely limited utility in identifying true cases of ventricular hypertrophy in this cohort. Sensitivity, specificity, PPV and NPV of R/S overlap for specific chest leads are presented in Table 1.

**Table 1:** Sensitivity and specificity of R/S overlap for specific chest leads.

Lead	Sensitivity	Specificity
V1/V2	28.6%	93.4%
V2/V3	52.2%	73.3%
V3/V4	69.2%	45.0%
V4/V5	47.8%	75.7%
V5/V6	8.7%	95.6%
	PPV	NPV
R/S OL	4.0%	99.0%

V1/V2, overlapping of R and S waves in chest leads V1 and V2; V2/V3, overlapping of R and S waves in chest leads V2 and V3; V3/V4, overlapping of R and S waves in chest leads V3 and V4; V4/V5, overlapping of R and S waves in chest leads V4 and V5; V5/V6, overlapping of R and S waves in chest leads V5 and V6; R/S OL, R/S olverlap; PPV, positive predictive value; NPV, negative predictive values.

**Figure 2:**
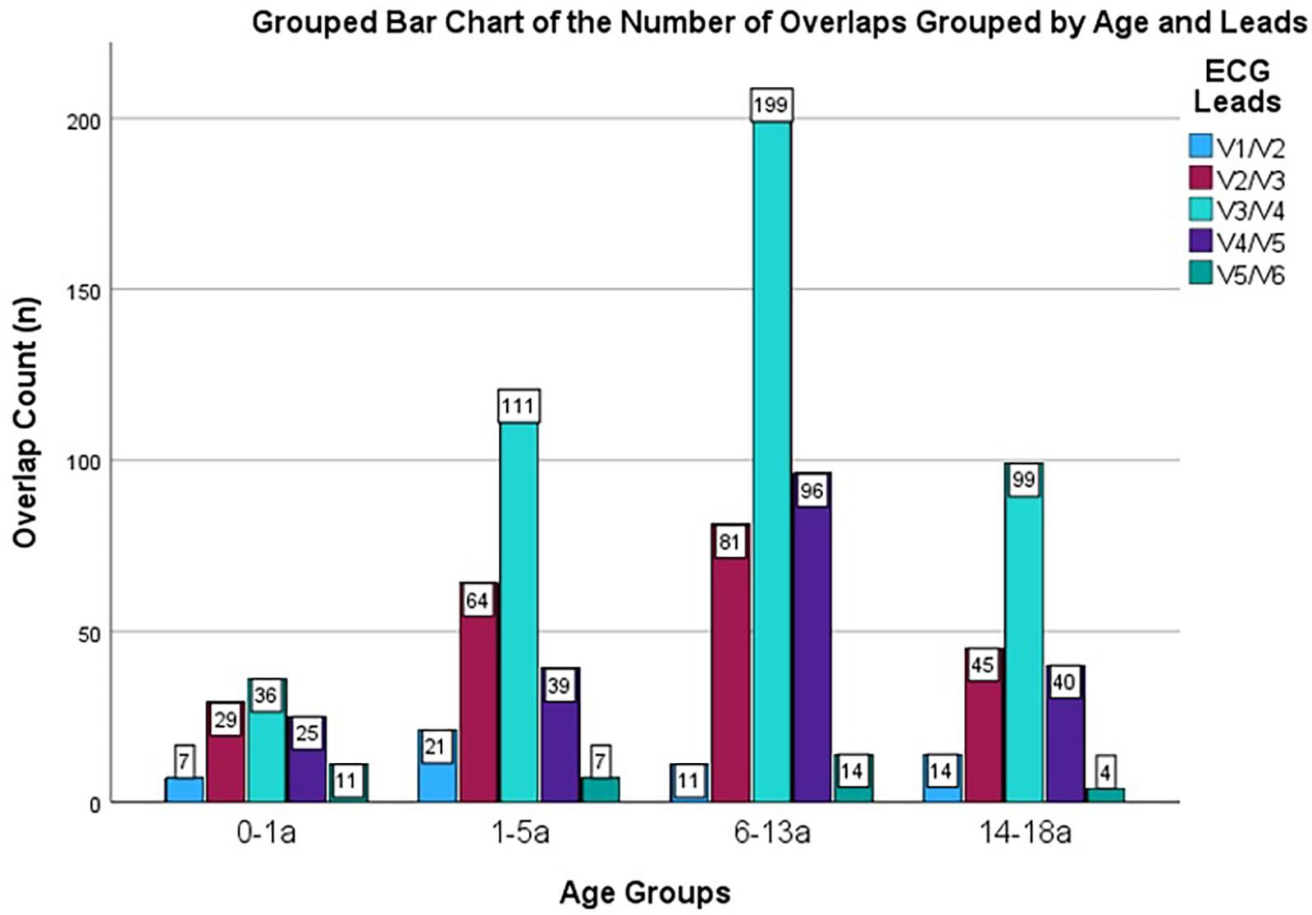
Distribution of R/S overlap among different age groups. a = years; n = number; age is given in years; color coding explained in right upper corner—displaying R/S overlap in V 1/2 etc. respectively.

R/S ratio and amplitudes of R and S waves among different age groups are presented in Tables 2, 3. Amplitudes of R and S waves were not analyzed in the non-overlap group. Distribution of R/S overlap among different age groups is presented in Figure 2.

**Table 2:** R/S ratio in chest leads among different age groups.

Age group	V1	V2	V3	V4	V5	V6
Newborns (0–12 mo)	1.4 (0.0–21.5)	1.1 (0.0–8.3)	1.1 (0.0–5.2)	1.4 (0.1–13.0)	2.0 (0.2–13.7)	2.3 (0.4–22.0)
Children (1–5 y)	0.5 (0.0–12.8)	0.6 (0.0–13.8)	0.7 (0.0–16.3)	2.4 (0.2–105.0)	4.3 (0.1–45.0)	5.5 (0.2–50.0)
Children (6–18 y)	0.3 (0.0–25.0)	0.4 (0.0–17.0)	0.5 (0.0–38.8)	2.4 (0.0–105.0)	5.3 (0.02–210.0)	7.5 (0.0–55.0)

R/S ratios are given as median (range). mo, months; y, years.

**Table 3:** Amplitudes of R and S waves among different age groups.

Age group	Lead	R wave	S wave
Newborns (0–12 mo)	V1	0.6 (0.1–2.2)	0.5 (0.1–1.7)
	V2	1.1 (0.1–2.1)	1.0 (0.0–3.1)
	V3	1.3 (0.0–2.7)	1.2 (0.5–2.7)
	V4	1.4 (0.1–2.7)	1.1 (0.1–2.3)
	V5	1.2 (2.4–2.7)	0.8 (0.0–2.1)
	V6	0.9 (0.4–1.8)	0.5 (0.0–1.8)
Children (1–5 y)	V1	0.3 (0.0–3.9)	0.6 (0.0–4.1)
	V2	0.8 (0.01–3.5)	1.2 (0.0–4.1)
	V3	1.1 (0.1–3.3)	1.4 (0.2–11.5)
	V4	1.4 (0.2–2.9)	0.7 (0.0–2.5)
	V5	1.3 (0.02–2.8)	0.3 (0.0–1.6)
	V6	0.9 (0.1–2.3)	0.1 (0.0–1.1)
Children (6–18 y)	V1	0.3 (0.0–2.5)	0.7 (0.0–3.7)
	V2	0.5 (0.0–6.5)	1.1 (0.0–3.7)
	V3	0.8 (0.0–3.6)	1.4 (0.0–5.1)
	V4	1.5 (0.0–4.5)	0.5 (0.0–3.9)
	V5	1.4 (0.1–4.3)	0.2 (0.0–2.7)
	V6	1.1 (0.0–3.0)	0.1 (0.0–1.5)

R and S wave amplitudes are given as median (range). mo, months; y, years.

**Figure 3:**
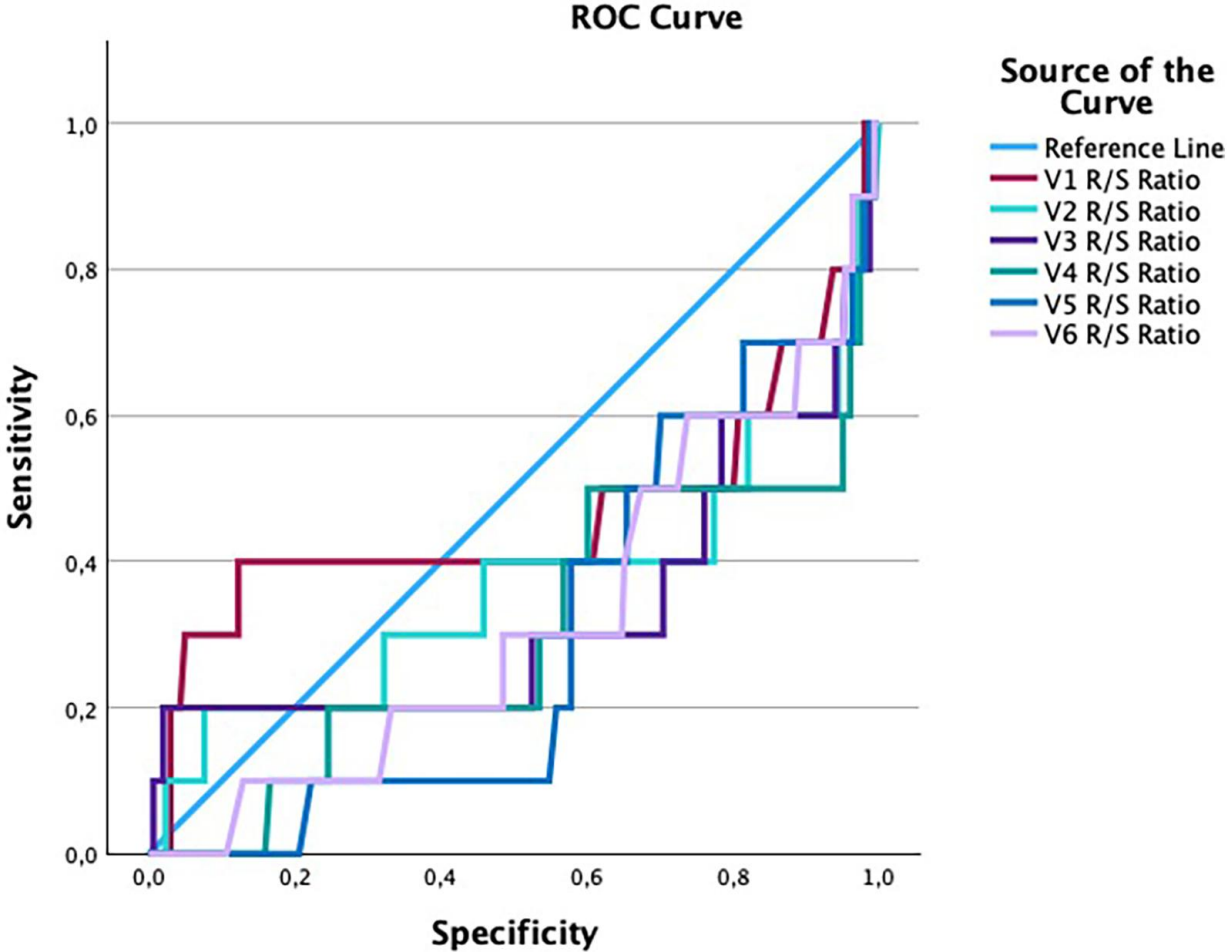
ROC curves of R/S ratios in chest leads V1–V6. Reference line = blue; color coding is given in right upper corner.

The AUC values for the R/S ratios in leads V1–V6 ranged from 0.30 to 0.46, all below the reference value of 0.5. None reached statistical significance (*p* > 0.05). ROC curves are illustrated in Figure 3, AUC values are presented in Table 4. The wide 95% confidence intervals and sub-random AUC values indicate that the R/S ratio does not provide meaningful discriminatory power for detecting cardiac hypertrophy in our cohort.

**Table 4:** Area under the ROC curve (AUC) for R/S ratios by chest lead.

Test Result Variable	AUC	Std. Error	Asymptotic Sig.	95% CI Lower Bound	95% CI Upper Bound
V1 R/S ratio	0.46	0.13	0.76	0.21	0.71
V2 R/S ratio	0.37	0.12	0.24	0.14	0.59
V3 R/S ratio	0.33	0.11	0.14	0.11	0.55
V4 R/s ratio	0.30	0.10	0.04	0.11	0.49
V5 R/S ratio	0.30	0.07	0.01	0.15	0.44
V6 R/S ratio	0.32	0.09	0.05	0.15	0.50

AUC, area under the curve; Std., standard; Sig., significance.

A total of two patients (0.25%) with biventricular cardiac hypertrophy were identified. None of these patients met the Katz-Wachtel criteria of high R and S waves (≥50 mm). Five male patients (0.63%) without confirmed cardiac hypertrophy exhibited positive Katz-Wachtel sign (lead V2 = 1 patient; lead V3 = 4 patients).

### Statistical analysis

Chi-square tests demonstrated a statistically significant, albeit weak, correlation between the presence of R/S overlap in chest leads and cardiac hypertrophy (*p* < 0.05). The strength of the correlation between R/S overlap and cardiac hypertrophy was determined using Phi and Cramer's *V* values. Phi value: −0.087 (indicating a weak correlation). Cramer's *V* value: 0.087 (consistent with a weak association).

Most R/S overlaps were observed in leads V3/V4, irrespective of age group and hypertrophy status. Children with cardiac hypertrophy exhibited R/S overlap more often in leads V2/V3 and V4/V5, compared to children without myocardial hypertrophy.

Inter-observer agreement for manual ECG measurements by two independent pediatric cardiologists showed intraclass correlation coefficients (ICC) between 0.86 and 0.91. For binary classifications (presence of R/S overlap and Katz-Wachtel phenomenon). Inter-rater agreement assessed using Cohen's *κ* showed values of 0.82 and 0.86, respectively, indicating substantial agreement.

## Discussion

This is the first study evaluating R/S overlap in chest leads and KWP in a large pediatric population. The following key findings of our study highlight the clinical relevance of R/S overlap in chest leads in the pediatric population:

1. **Higher Prevalence of Cardiac Hypertrophy in R/S overlap:** Cardiac hypertrophy was present in 4% of patients with R/S overlap compared to 1% in those without, indicating a higher prevalence of ventricular hypertrophy in the R/S overlap group.
2. **Sensitivity and Specificity:** R/S overlap demonstrated moderate sensitivity (max. 69%) but high specificity (up to 95%) for detecting cardiac hypertrophy. The highest sensitivity was observed in leads V3/V4 (69%), while specificity was highest in leads V1/V2 (93%) and V5/V6 (95%), respectively.
3. **R/S Ratio and Amplitudes:** The mean R/S ratio was elevated in infants irrespective of hypertrophy status. Older children with hypertrophy exhibited higher R/S ratios, whereas older children without hypertrophy showed lower ratios. Additionally, hypertrophic patients had lower R and S wave amplitudes in leads V2/V3 and V3/V4 and higher amplitudes in V4/V5 and V5/V6.
4. **Katz-Wachtel Phenomenon:** None of the patients with cardiac hypertrophy met the Katz-Wachtel criteria, while five non-hypertrophic patients fulfilled Katz-Wachtel criteria.

### Interpretation of results

Our analysis demonstrates that while R/S overlap exhibits high sensitivity in detecting pediatric ventricular hypertrophy, its poor specificity significantly limits its utility as a standalone diagnostic tool. The high rate of false positives suggests that R/S overlap alone should not be used to confirm hypertrophy and must be interpreted in conjunction with echocardiographic findings and other clinical parameters. This reinforces the need for more comprehensive diagnostic approaches when assessing pediatric patients for hypertrophy-related conditions.

### Interpretation in the context of multicriteria ECG analysis

It is important to acknowledge that clinical ECG interpretation for ventricular hypertrophy relies not on isolated findings, but on a combination of multiple criteria—such as voltage thresholds, repolarization abnormalities, axis deviation, and chamber enlargement patterns. The present study does not advocate for the use of R/S overlap or the Katz-Wachtel phenomenon as standalone diagnostic tools. Rather, our objective was to assess the individual diagnostic performance of these historically referenced features to better understand their relative contribution within a multimodal interpretive framework. This focused evaluation allows clinicians to critically appraise the diagnostic weight of specific signs and supports the broader movement toward evidence-based refinement of pediatric ECG interpretation.

An unexpected finding in our study was that the Katz-Wachtel phenomenon did not correlate with confirmed cases of hypertrophy but was observed in patients without echocardiographic evidence of ventricular enlargement. This challenges the long-held assumption that Katz-Wachtel is a reliable indicator of hypertrophy in pediatric patients. Given this discrepancy, our findings suggest that the traditional definition of the Katz-Wachtel phenomenon may require reconsideration.

The overall results highlight the need for caution in over-reliance on historical ECG markers for diagnosing pediatric hypertrophy. While R/S overlap may serve as a useful screening tool due to its sensitivity, it should not be solely relied upon in clinical decision-making. Furthermore, our findings regarding the Katz-Wachtel phenomenon underscore the necessity of reassessing classical ECG criteria within the modern pediatric population. The lack of diagnostic value of the Katz-Wachtel phenomenon in this study contrasts with earlier literature that described it as a significant marker for biventricular hypertrophy (5). This may be attributed to the larger number of children included in this study, or the incorporation of echocardiographic criteria for cardiac hypertrophy. However, the use of advanced ECG recording technology, applying digital recording and filtering may also impact the results. Historical ECG criteria, such as the KWP, should generally be used with caution and be subject to reevaluation in light of modern ECG recording technology.

### Impact of digital ECG processing

Modern ECG systems apply a range of digital processing techniques—including baseline filtering, amplitude normalization, and dynamic scaling—to optimize signal clarity and suppress noise. While these features improve general readability, they may inadvertently alter the true morphology and amplitude of QRS complexes, particularly in pediatric patients with low-voltage or high-frequency signals. In this context, the apparent overlap of R and S waves (R/S overlap) may be accentuated or diminished depending on filtering strength and lead-specific gain settings. More notably, the Katz-Wachtel phenomenon—which is defined by exceptionally high-amplitude biphasic QRS complexes—may be masked by automated amplitude compression or normalization algorithms. This technological shift likely contributes to the limited prevalence of the Katz-Wachtel sign in our cohort and supports the argument that historical criteria derived from analog recordings should be re-evaluated in light of modern ECG technology.

As discussed in the literature (2, 8), further research, ideally incorporating larger, multicenter cohorts, will be critical in determining whether these markers retain any diagnostic relevance or should be revised.

The variations in R/S ratio across different age groups highlight the importance of considering age-specific normal ranges when interpreting pediatric ECGs (14). The elevated R/S ratio in infants reflects normal physiological changes, while the differences observed in older children with and without hypertrophy underscore the need for age-adjusted diagnostic criteria.

The differences in R and S wave amplitudes between hypertrophic and non-hypertrophic patients in specific leads (V2/V3 and V4/V5) suggest that amplitude measurements could complement R/S overlap in identifying cardiac hypertrophy. The lower amplitudes in leads V2/V3 and V3/V4 and higher amplitudes in V4/V5 and V5/V6 among hypertrophic patients may reflect the altered ventricular mass and electrical activity distribution.

The findings of this study are consistent with previous research that identified R/S overlap as a common feature in pediatric ECGs but questioned its specificity for diagnosing cardiac hypertrophy (10). Studies have reported varying prevalence rates and diagnostic accuracy, reflecting differences in study populations, methodologies, and diagnostic criteria (2, 10).

The weak correlation between R/S overlap and cardiac hypertrophy aligns with earlier reports suggesting that R/S overlap should be interpreted in the context of other clinical and diagnostic findings (2). The high sensitivity observed in this study corroborates findings from studies emphasizing the utility of R/S overlap as a screening tool, particularly in resource-limited settings where advanced imaging modalities may not be readily available.

### Clinical implications

Given the high sensitivity but low specificity of R/S overlap, we recommend that it be considered as a preliminary screening tool rather than a definitive diagnostic criterion for ventricular hypertrophy in pediatric patients. Its presence should prompt further evaluation, particularly echocardiography, in cases where additional risk factors for hypertrophy exist (e.g., clinical suspicion, family history, or abnormal cardiac examination findings). Pediatricians and cardiologists should exercise caution in over-reliance on this parameter and incorporate additional clinical and imaging data to support a diagnosis.

Based on our findings, we propose the following decision framework for clinicians:

- When should R/S overlap prompt further evaluation?
  ○ If detected in a pediatric ECG, clinicians should assess additional risk factors such as: presence of clinical symptoms (e.g., dyspnea, fatigue, exercise intolerance), family history of cardiomyopathy or congenital heart disease, and abnormal findings on cardiac examination (e.g., murmur, gallop rhythm, hepatomegaly). If risk factors are present, echocardiography should be performed.

In summary, while ECG remains a valuable tool in pediatric cardiology, traditional markers such as R/S overlap and the Katz-Wachtel phenomenon require critical reassessment. Our findings emphasize the need for a more nuanced approach to ECG interpretation, incorporating multimodal assessments to ensure accurate and reliable hypertrophy diagnosis in pediatric patients.

### Study limitations

Manual measurement of ECG parameters, while precise, may be subject to inter-observer variability. To mitigate this, all measurements were performed by two independent observers, and discrepancies were resolved through consensus. Future studies could benefit from automated ECG analysis to enhance measurement accuracy and reproducibility.

We acknowledge that the overall prevalence of ventricular hypertrophy in our cohort was low (2.9%), which limits the statistical power to draw definitive conclusions about the sensitivity and specificity of the ECG criteria analyzed. This limitation is particularly relevant for rare findings such as the Katz-Wachtel phenomenon, where small case numbers increase the risk of type II error. As this was a retrospective, exploratory analysis based on all eligible patients over a six-year period, no formal *a priori* sample size calculation was performed. Future prospective studies should consider targeted recruitment or enrichment strategies to ensure adequate representation of ventricular hypertrophy cases and to allow for robust estimation of diagnostic performance with appropriate statistical power.

This study was conducted at a single tertiary care center, which may limit the generalizability of the findings to broader pediatric populations. Multicenter studies involving diverse clinical settings are warranted to validate and extend these results.

## Conclusion

This study highlights the association between R/S overlap in chest leads and cardiac hypertrophy in pediatric patients. The findings suggest that R/S overlap can serve as a useful screening tool, but its low specificity necessitates further diagnostic evaluations. The Katz-Wachtel phenomenon lacks diagnostic value. It´s use in the pediatric population should be discontinued. Ultimately, a comprehensive diagnostic approach, incorporating age-specific criteria and combining ECG with echocardiography, is essential for accurately diagnosing and managing cardiac hypertrophy in pediatric patients.

## Data Availability

The raw data supporting the conclusions of this article will be made available by the authors, without undue reservation.
